# A novel pathogenic large germline deletion in adenomatous polyposis coli gene in a Chinese family with familial adenomatous polyposis

**DOI:** 10.18632/oncotarget.10408

**Published:** 2016-07-06

**Authors:** Zhao Zhang, Shengran Liang, Hui Huang, Dan Wang, Xipeng Zhang, Jing Wu, Huishuang Chen, Yanyan Wang, Tingting Rong, Yulin Zhou, Santasree Banerjee

**Affiliations:** ^1^ Tianjin University of Traditional Chinese Medicine, Department of Colorectal Surgery, Tianjin Union Medical Center, Tianjin 300121, China; ^2^ BGI-Shenzhen, Shenzhen 518083, China; ^3^ Department of Pathology, Tianjin Medical University General Hospital, Tianjin 300000, China; ^4^ Xiamen Prenatal Diagnosis Center, Xiamen Maternal and Child Health Care Hospital, Xiamen 361000, China

**Keywords:** familial adenomatous polyposis, APC gene, large exon deletion, targeted next-generation sequencing, colorectal cancer

## Abstract

Germline mutations of the *APC* gene are associated with an autosomal dominant precancerous condition, termed familial adenomatous polyposis (FAP). FAP is clinically manifested by the presence of multiple colorectal adenomas or polyps. Gradually, these colorectal adenomas or polyps inevitably result in colorectal cancer by the third-to fourth decade of life. Surgical interventions or total proctocolectomy is the best possible treatment for FAP. Here, we present a clinical molecular study of a five generation Chinese family with FAP. Diagnosis of FAP was made on the basis of clinical manifestations, family history and medical (colonoscopy and histopathology) records. Blood samples were collected and genomic DNA was extracted. Genetic screening of the *APC* gene was performed by targeted next-generation sequencing and quantitative real-time PCR. Targeted next generation sequencing identified a novel heterozygous large deletion [exon5-exon16; c.423_8532del] of *APC* gene, which segregated with the FAP phenotypes in the proband and in all the affected family members. Unaffected family members and normal controls did not carry this deletion. In the Chinese population, most of the previously reported *APC* gene mutations are missense mutations. This is the first report describing the largest deletion of the *APC* gene in the Chinese population associated with FAP.

## INTRODUCTION

Familial adenomatous polyposis (FAP) [MIM# 175100] is a familial precancerous condition characterized by the presence of numerous colorectal adenomas or polyps, with an incidence of 3-10/100,000 [1]. It is an autosomal dominant colon cancer predisposition syndrome accounting for approximately 1% of all colorectal cancers (CRC). FAP invariably results in colorectal cancer (CRC), if not detected and diagnosed early and the colon removed by total proctocolectomy [2]. Colorectal adenomas usually start to appear at the third decade of life and if untreated, gradually become symptomatic with presence of numerous colorectal adenomas or polyps by the fourth decade of life [3]. Apart from colorectal adenomas, extracolonic manifestations such as desmoids tumors, osteomas, dental abnormalities, congenital hypertrophy of the retinal pigment epithelium (CHRPE), lipomas, epidermoid cysts and upper gastrointestinal polyps may also develop in patients with FAP [4]. In addition, it has been found that FAP is also associated with thyroid cancer, brain cancer and cancers of hepatobiliary tract. On the basis of the number of colorectal adenomas or polyps and the age of onset of the patient, FAP is categorized into two types, namely; the classical FAP (CFAP) and the attenuated FAP (AFAP) [5]. Patients with CFAP usually manifest more than 100 colorectal adenomatous polyps, generally start to develop at the third decade of the life. Patients with AFAP characteristically have 10–100 colorectal adenomatous polyps, generally start to develop at the fourth decade of life. [6].

Germline mutations of the *APC* gene are associated with FAP. *APC* is a tumor suppressor gene [7, 8], associated with cell adhesion, transcriptional activation, cell migration, and apoptosis [9, 10]. The APC protein is a multi-functional molecule comprises of eight known functional subdomains involved in the regulation of cell adhesion, polarization, and migration. The main function of the APC protein is to regulate the β-catenin protein level. Mutations of *APC* result in the accumulation of the β-catenin protein in cytoplasm through the activation of other transcription factors including Tcf, which in turn causes aberrant activation of the canonical *Wnt* signaling pathway leading to uncontrolled cell proliferation, progression and development of colon cancer [11]. According to the previous published reports, the majority of pathogenic *APC* germline mutations belong to three categories, nonsense/frameshift mutations, splice sites mutations and deep intronic deletions. Nonsense/frameshift mutations splice sites mutations and deep intronic deletions of the *APC* gene lead to large genomic rearrangements, resulting in the formation of truncated APC proteins [12]. Although, it has been found that in few cases, point mutations or missense variants within the coding sequence also result in the formation of alternative transcripts due to aberrant splicing [13, 14]. It has been reported that 2% of all germline *APC* gene mutations are large genomic deletions [15]. Moreover, in HGMD, 1000 different *APC* germline mutations have been reported till date (http://www.hgmd.cf.ac.uk/ac). In addition, over 194 unique *APC* germline mutations from 191 patients have been reported in the Chinese population with 76 small deletions (75 small deletions are in coding region and 1 deletion in intron) (http://www.genomed.org/lovd2/home.php?select_db=APC). In the Chinese population, there was no large deletion of APC gene has previously been reported. However, the 5′end of the *APC* gene appears to be the most common site for germline mutations. The codon 1309 of the *APC* gene is a mutation cluster region (MCR) with a higher frequency of *APC* germline mutations. The location of the mutation in the *APC* gene is very significant as it is directly correlated with the phenotypic spectrum of the disease, age of onset and the appearance of extracolonic manifestations in FAP patients [16].

Here, in order to identify the molecular basis of FAP in the proband and in all the affected members of this five generation Chinese family, we screened a panel of 14 genes (*APC, MLH1, MSH2, MSH6, PMS2, AXIN2, BMPR1A, EPCAM, MLH3, MUTYH, PMS1, PTEN, SMAD4, STK11*) associated with colorectal cancer by targeted next-generation sequencing and Quantitative Real-Time PCR (qPCR). We identified a novel heterozygous large germline deletion of *APC* gene segregating with FAP phenotype among all the FAP patients in this five generation Chinese family, with autosomal dominant inheritance.

## RESULTS

### Family recruitment and clinical examination

**Figure 1:**
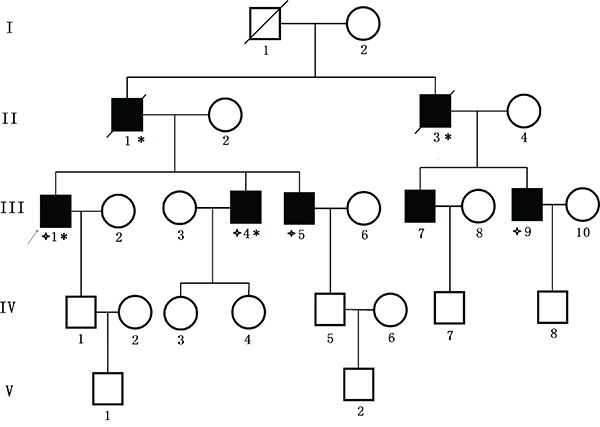
Pedigree structure of the Chinese family with familial adenomatous polyposis Family members with FAP are indicated with Shading. Squares and circles denoted males and females respectively. Individuals labeled with a solidus were deceased. Family members with CRC are indicated with asterisks. Family members detected with APC gene deletion are indicated by “four angled star”. Roman numerals indicate generations. Arrow indicates the proband (III-1).

**Figure 2:**
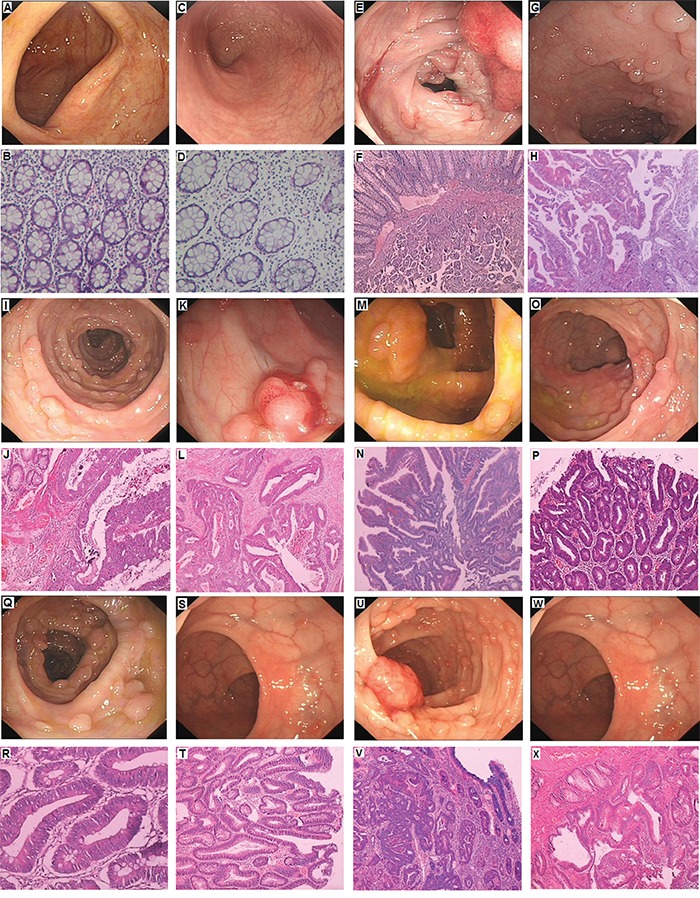
Clinical Description **A-B.** No polyps in the colon of unaffected member with normal histology (III-2). **C-D.** No polyps in the rectum of unaffected member with normal histology (III-2). **E-F.** Polyps in the colon, Cancer invading in the submucosa in proband (III-1); **G-H.** Polyps of rectum showing Low grade intraepithelial neoplasia (dysplasia), the gland of local lesion showing High grade intraepithelial neoplasia (dysplasia) in proband (III-1). **I-J.** Polyps of colon showing High grade intraepithelial neoplasia (dysplasia) in III-4; **K-L.** Polyps of rectum, adenocarcinoma in III-4. **M-N.** Polyps of colon is Low grade intraepithelial neoplasia (dysplasia), the gland of local lesion is High grade intraepithelial neoplasia (dysplasia) in III-5; **O-P.** Polyps of rectum are Low grade intraepithelial neoplasia (dysplasia) in III-5; **Q-R.** Polyps of colon are Low grade intraepithelial neoplasia (dysplasia) in III-7; **S-T.** Polyps of rectum are Low grade intraepithelial neoplasia (dysplasia) in III-7; **U-V.** Polyps of colon are High grade intraepithelial neoplasia (dysplasia) in III-9; **W-X.** Polyps of rectum are Low grade intraepithelial neoplasia (dysplasia) in III-9.

We identified a five generation Chinese pedigree with 23 members, among whom five individuals were affected by FAP including two with CRC (Figure 1). Another 2 affected family members (II-1 and II-3) had died from CRC. In Table 1, we have described the detailed and comprehensive clinical information for all the affected and unaffected members in this family. A comprehensive and comparative colonoscopy and histopathology of colon and rectum for all the affected family members along with an unaffected member (III-2) are shown in Figure 2.

**Table 1:** Clinical characteristics of all the affected and unaffected family members found in our study

Family ID	Sex	Exon Deletion	Present Age (Years)	Age at Diagnosis	No. of Polyps	CRC Age	Extra-colonic Features
**I-1**	M	-	Died (73)	-	-	-	-
**I-2**	F	WT	70	-	-	-	-
**II-1**	M	-	Died (69)	-	-	-	-
**II-2**	F	WT	76	-	-	-	-
**II-3**	M	-	Died (56)	-	-	-	-
**II-4**	F	WT	70	-	-	-	-
**III-1**	M	Del	53	50	~1000	53	-
**III-2**	F	WT	52	-	-	-	-
**III-3**	F	WT	51	-	-	52	-
**III-4**	M	Del	50	49	~1000	-	-
**III-5**	M	Del	48	46	~500	-	-
**III-6**	F	WT	48	-	-	-	-
**III-7**	M	(Sample Unavailable)	42	42	~300	-	-
**III-8**	F	WT	40	-	-	-	-
**III-9**	M	Del	38	38	>100	-	-
**III-10**	F	WT	37	-	-	-	-
**IV-1**	M	-	36	-	-	-	-
**IV-2**	F	-	35	-	-	-	-
**IV-3**	F	-	27	-	-	-	-
**IV-4**	F	-	14	-	-	-	-
**IV-5**	M	-	27	-	-	-	-
**IV-6**	F	-	26	-	-	-	-
**IV-7**	M	-	6	-	-	-	-
**IV-8**	M	-	17	-	-	-	-
**V-1**	M	-	5	-	-	-	-
**V-2**	M	-	1	-	-	-	-

### Identification and characterization of candidate mutation

A heterozygous novel large deletion; [exon5-exon16; c.423_8532del]in *APC* gene [NCBI Reference sequence NM_000038.3] was identified in proband (III-1) by targeted next generation sequencing. This heterozygous novel large deletion co-segregated with the FAP phenotypes in the proband (III-1) and amongst all the affected family (III-4, III-5 and III-9) members, (sample of III-7 was unavailable) but absent in the unaffected family members. We did not detect this mutation in the normal control of the same ethnic origin, gender and age range.

### Confirmation of the novel large deletion by quantitative real-time PCR (qPCR)

**Figure 3:**
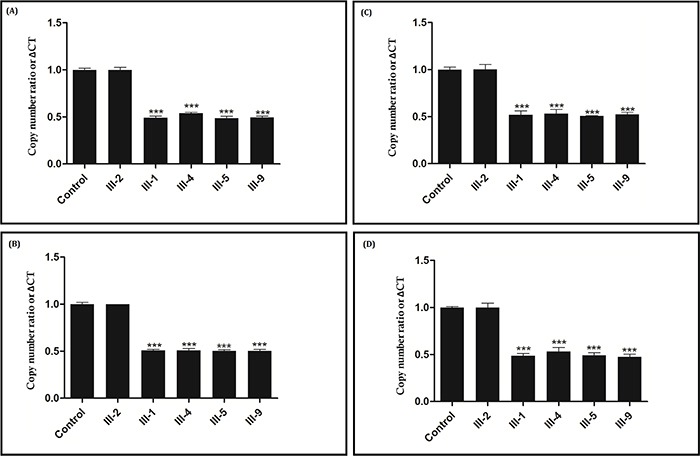
Verification of the novel heterozygous large deletion in III-1, III-4, III-5 and III-9 by quantitative real-time PCR (qPCR) Copy number ratio of the exon 5 **Figure 3A.**, exon 9 **Figure 3B.**, exon 12 **Figure 3C.** and exon 16 **Figure 3D.** in THE control and in A normal family member (III-2) is nearly 1, and the Copy number ratio in proband (III-1) and other affected family members (III-4, III-5 and III-9) is approximately half. Actin DNA was used as a loading control.

The relative DNA copy number for the *APC* 5, 9, 12, 16 exons was measured by quantitative real-time PCR (qPCR) in the proband (III-1) and in all the affected family members (III-4, III-5 and III-9) (Sample of III-7 was unavailable). As this is a large deletion, for validation of the result, qPCR was undertaken in the proband (III-1) and in all affected family members [III-4, III-5 and III-9], an unaffected family member (III-2) with actin being used as a control [sample of III-7 was unavailable]. For exon5, a normal negative control and unaffected family members (III-2) gave an amplification level of 1.0, while the relative amplification from the proband (III-1) and in all the affected family members [III-4, III-5 and III-9] was approximately 0.48 (Figure 3A). For exon9, a normal negative control and unaffected family members (III-2) gave an amplification level of 1.0, but the relative amplification from the proband (III-1) and in all the affected family members [III-4, III-5 and III-9] was approximately 0.5 (Figure 3B). For exon12, a normal negative control and unaffected family members (III-2) gave an amplification level of 1.0, while the relative amplification from the proband (III-1) and in all the affected family members [III-4, III-5 and III-9] was approximately 0.51 (Figure 3C). For exon16, a normal negative control and unaffected family members (III-2) gave an amplification level of 1.0, but the relative amplification from the proband (III-1) and in all the affected family members [III-4, III-5 and III-9] was approximately 0.48 (Figure 3D). There was a significant difference between affected family members [III-1 (proband), III-4, III-5 and III-9], unaffected family members (III-2) and normal control, suggesting that there was a heterozygous deletion of exon5-16 of *APC* in the proband (III-1) and in all the affected family members (III-4, III-5and III-9). Hence, q-PCR data suggest that proband (III-1) and affected family members (III-4, III-5 and III-9) have a novel heterozygous deletion of exon5-16 of the *APC* gene that is associated with FAP in this family.

## DISCUSSION

**Figure 4:**
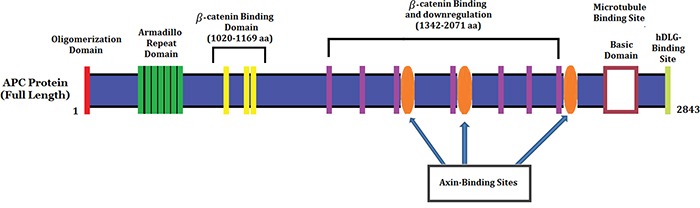
Schematic Diagram of the APC protein structure with functional domains

In our study, we found a heterozygous novel large deletion (exon5-exon16; c.423_8532del) [NCBI Reference sequence NM_000038.3] of the human *APC* gene in the proband (III-1) and among all the FAP-affected family members [III-4, III-5 and III-9] in a five generation Chinese family. This heterozygous novel large deletion of *APC* gene results in the formation of truncated APC protein with complete loss of the armadillo repeat domain,β-catenin binding site, microtubule binding site, EBI Domain, hDLG binding site and PDZ binding domain (Figure 4). This heterozygous novel large deletion of *APC* gene is not present in the ExAC database.

In this Chinese family, diagnosis of FAP has been done according to a review of clinical report, endoscopic and histo-pathological data. However, the presence of specific clinical symptoms (colorectal adenomas/polyposis) and the autosomal dominant mode of inheritance allow the diagnosis of the disease in this family as FAP. In our study, the heterozygous novel large deletion of *APC* gene in this Chinese family manifests with colorectal adenomas (polyps) without any extra-colonic manifestations.

Large deletions of individual genes or single exon or several exons represent a well-known molecular basis of hereditary nonpolyposis colorectal cancer (HNPCC) [17, 18]. However, only 47 whole *APC* gene deletions have been published. In addition, most of the cases with whole *APC* gene deletions were diagnosed as classic FAP. Moreover, in only one case with a whole *APC* gene deletion found to be associated with attenuated phenotype [19]. Until now, 43 partial *APC* gene deletions have been reported to be associated with FAP. Among all the partial deletion of *APC* exons cases, deletions of exons 14 (6 probands), 14–15 (5 probands), 15 (4 probands), 8–15 (4 probands) and 9–15 (4 probands) are the most frequent. The majority of the cases with partial *APC* gene deletions were also associated with classic FAP. Partial deletions of *APC* gene are of either *in-frame* or *out-of-frame*, which correlates with the phenotype of FAP patients. *APC* gene *in-frame* deletions result in the formation of a shorter but possibly functional APC protein, so the resulting phenotype of the FAP is milder. The previously reported *in-frame* or *out-of-frame* deletions of *APC* gene never include the promoter region and exon 15 because the promoter region of *APC* gene is important for initiation of transcription and exon 15 is necessary for regulating the turnover and localization of β-catenin [20, 21].

### APC gene mutation

A large number of pathogenic *APC* gene mutations have been reported in different countries and ethnic groups [16]. According to the HGMD dataset, small deletions account for the majority of *APC* gene mutations, resulting in alteration of the open reading frame followed by the formation of a truncated gene product. Until now, 275 sequence variants of the *APC* gene have been reported in the Chinese population. Among these, 194 are unique variants were identified out of 191 individuals. Among the 275 sequence variants of the APC gene, the majority is substitutions (164), but frameshift (101), nonsense (83), deletion (76), insertion (33) and inversion (2) are also reported (http://www.genomed.org/lovd2/variants_statistics.php).

Previous reports had not described large deletions of the *APC* gene in the Chinese population (http://www.genomed.org/lovd2/variants.php?select_db=APC&action=view_all&view=DNA_del).

According to a very recent report, a novel deletion in exon 14–15(c.1936-2148del) and intron 14 was described as causing FAP without any extracolonic manifestation in a Chinese family [1].

### Genotype–phenotype correlation

Genotype–phenotype correlation studies are very significant and enable us to define the most likely phenotype to be associated with a given mutation. The identification and characterization of *APC* mutation carriers with a well diagnosed phenotype will allow us to establish specific surveillance programs and prophylactic surgical treatment. *APC* germline mutations, affecting the mutation cluster region (MCR), are correlated with more severe phenotype and early age of onset in FAP patients [23–27].

In conclusion, the present study describes a heterozygous novel large deletion mutation in *APC* gene in a five generation Chinese family with FAP. Our study expands the spectrum of the germline mutations of *APC* gene in the Chinese population. This novel finding contributes to a more comprehensive database of germline mutations of *APC* gene that could be used for the molecular diagnosis, risk assessment, susceptibility of the disease for the FAP patients.

## MATERIALS AND METHODS

### Ethical statement

Family members of this five generation Chinese family have given written informed consent as they are participating in this study. The Ethical Committee of the Tianjin Union Medical Center, China, reviewed and approved our study protocol in compliance with the Helsinki declaration. Diagnosis of the patients for FAP has done by oncologists, on the basis of clinical test reports and detailed family pedigree.

### Patients and pedigree

A five generation Chinese family with FAP (Figure 1), diagnosed and treated in the Department of Colorectal Surgery, Tianjin Union Medical Center, 300121, China, were enrolled in our study. Clinical diagnosis of FAP was established in this family by endoscopic screening after the proband (III-1) presented to Tianjin Union Medical Center with CRC. The diagnostic standard or criteria for patients with FAP was as follows: (1) patients having >100 colorectal adenomas or polyps and (2) at least 20 synchronous colorectal adenomas or polyps in patients with a positive family history of FAP.

### Targeted exome-based next-generation sequencing and variant identification

DNA samples obtained from the proband (III-1) were sequenced using target exome-based next-generation sequencing. Roche NimbleGen's (Madison, USA) custom Sequence Capture Human Array was used to designed to capture 98480 kb of targeted sequence, covering 181 exons and flanking sequence (including the 100 bp of introns) of 14 genes (*APC, MLH1, MSH2, MSH6, PMS2, AXIN2, BMPR1A, EPCAM, MLH3, MUTYH, PMS1, PTEN, SMAD4, STK11*) which is associated colorectal cancer (CRC) and yielded an average of 6366534 reads per sample, with approximately 68.78% mapping to the targeted regions. The average sequencing depth of the target area is 464.68% with 99.46% coverage. The procedure for preparation of libraries was consistent with standard operating protocols published previously. In each pooling batch, 10 to 33 samples were sequenced simultaneously on Illumina HiSeq 2500 Analyzers (Illumina, San Diego, USA) for 90 cycles (specially designed by us for this study). Image analysis, error estimation, and base calling were performed using Illumina Pipeline software (version 1.3.4) to generate raw data. The raw reads were screened to generate – clean reads'- followed by established filtering criteria. Clean reads with a length of 90 bp were aligned to the reference human genome from the NCBI database (Build 37) using the Burrows Wheeler Aligner (BWA) Multi-Vision software package with output files in - bam‖ format. The bamdata were used for reads coverage in the target region and sequencing depth computation, SNP and INDEL calling, and CNV detection. First, a novel three-step computational frame work for CNV was applied. Then, SNPs and INDELs were called using SOAPsnp software and Sam tools pileup software, respectively. A SNP or INDEL was be filtered if it could not follow the criterion: supported by at least 10 reads and >20% of the total reads. The frequency filter was set at 0.05. If a SNP frequency was more than 0.05 in any of the four databases (dbSNP, Hapmap, 1000 Genomes Project, the 124 healthy reference samples sequenced in this study), it would be regarded as a polymorphism, but not a causative mutation.

Last, SNVs were retrieved in The Human Gene Mutation Database (http://www.hgmd.cf.ac.uk/ac/index.php) and the Leiden Open Variation Database (http://www.lovd.nl/3.0/home), and then labeled as reported or novel.

### Quantitative real-time PCR (qPCR)

In order to validate the result of targeted next generation sequencing and further quantify the DNA copy number change for the *APC* gene q-PCR was undertaken. The relative DNA copy number for the *APC* 5, 9, 12, 16 exons were measured by quantitative real-time PCR (qPCR) using an ABI 7900HT Real-time PCR system (Life Technologies, Carlsbad, CA, USA) and HS qPCR Master Mix, according to the manufacturer's instructions. The primers used for amplifying *APC* exons were listed in Table 2.

**Table 2:** Detailed primer sequence for Real-time PCR

Primer	Primer sequence	Size/bp	Tm/°C
**Exon 5_Forward**	5′-TCATTGCTTCTTGCTGATCTTGAC-3′	74	59.8
**Exon 5_Reverse**	5′-GTGAGATTCTGAAGTTGAGCG-3′	74	57
**Exon 9_Forward**	5′-GGTTCAACTACACGAATGGACCATGA-3′	95	60
**Exon 9_Reverse**	5′-GTTCCCAGATGACTTGTCAGCCTTC-3′	95	60.2
**Exon 12_Forward**	5′-GTGGACTGTGAAATGTATGGGCT-3′	71	60.8
**Exon 12_Reverse**	5′-GCCATTCCAGCATATCGTCTTAG-3′	71	59.3
**Exon 16_Forward**	5′-ATAGGATGTAATCAGACGACACAGGAA-3′	120	58.5
**Exon 16_Reverse**	5′-CACTGCTGGAACTTCGCTCACA-3′	120	59.4

The PCR conditions were an initial denaturation step of 95°C for 10 min, followed by 95°C for 10 s, annealing (annealing temperature specific for a pair of primers) for 15 s and 72°C for 30 s, for a total of 45 cycles. The relative expression levels of *APC* were normalized to those of actin. The DNA copy number level for the *APC* exons (exon 5, 9, 12 and 16) in each sample were compared with the level in control blood samples from normal individual. Data were analyzed using the comparative threshold cycle (2–ΔΔCT) method.
